# Laparoscopic Cholecystectomy Using Indocyanine Green Fluorescence Imaging in a Patient with a Type I Accessory Hepatic Duct (Hisatsugu Classification): A Case Report

**DOI:** 10.70352/scrj.cr.25-0512

**Published:** 2025-10-28

**Authors:** Kohei Oyamada, Chikara Shirata, Taro Kataoka, Rioko Ide, Yuhei Oshima, Naganori Yamada, Kotaro Nishida, Makoto Hayasaka, Shinya Okata, Takayuki Okuno, Soichi Furukawa, Haruna Onoyama, Yasuaki Mochizuki, Hiroshi Kawasaki, Yusuke Kyoden

**Affiliations:** Department of Gastrointestinal and Vascular Surgery, Ibaraki Cancer Center, Ibaraki Prefectural Central Hospital, Kasama, Ibaraki, Japan

**Keywords:** laparoscopic cholecystectomy, accessory hepatic duct, Hisatsugu classification, indocyanine green fluorescence, biliary tract anomaly

## Abstract

**INTRODUCTION:**

Bile duct injury is a rare but serious complication of laparoscopic cholecystectomy (LC), particularly in patients with biliary anomalies such as accessory hepatic ducts (AHDs). Indocyanine green (ICG) fluorescence imaging has recently been recognized as a valuable tool for intraoperative visualization of biliary anatomy. However, its application in Type I AHDs, as classified by Hisatsugu, has not been previously reported.

**CASE PRESENTATION:**

A 35-year-old male was referred for elective LC following successful conservative treatment for mild acute cholecystitis. Preoperative magnetic resonance cholangiopancreatography revealed that the cystic duct drained into a posterior AHD arising from segment 6, consistent with a Type I anomaly according to the Hisatsugu classification. At anesthesia induction, 2.5 mg of ICG was administered intravenously, and near-infrared fluorescence imaging enabled clear identification of the biliary anatomy, including the AHD. Despite mild chronic inflammation, Calot’s triangle was safely dissected, and the gallbladder was successfully removed. Post-resection ICG imaging confirmed the integrity of the AHD, with no evidence of injury or stricture. The operative time was 3 h and 30 min, and the patient was discharged without complications on POD 3.

**CONCLUSIONS:**

Intraoperative ICG fluorescence imaging allowed for safe and accurate identification of biliary anatomy in a patient with a Type I AHD anomaly. This technique may help reduce the risk of bile duct injury during LC in patients with complex biliary variations.

## Abbreviations

AHD
: accessory hepatic duct
DIC-CT
: drip infusion cholangiography with CT
ICG
: indocyanine green
LC
: laparoscopic cholecystectomy
MRCP
: magnetic resonance cholangiopancreatography

## INTRODUCTION

LC is the standard surgical treatment for various gallbladder diseases and is among the most frequently performed procedures worldwide.^1)^ However, bile duct injury remains one of the most serious complications of this surgery, with a reported incidence of 0.2%–1.3%.^2)^ The risk is particularly high in patients with biliary tract anomalies.^3)^ ICG fluorescence imaging has emerged as a valuable tool in hepatobiliary and pancreatic surgery, particularly for intraoperative identification of bile ducts and liver tumors.^4)^ Here, we report a case of cholelithiasis associated with a rare biliary anomaly in which the cystic duct drained into an AHD. Intraoperative ICG fluorescence imaging enabled precise anatomical identification, allowing LC.

## CASE PRESENTATION

**Fig. 1:**
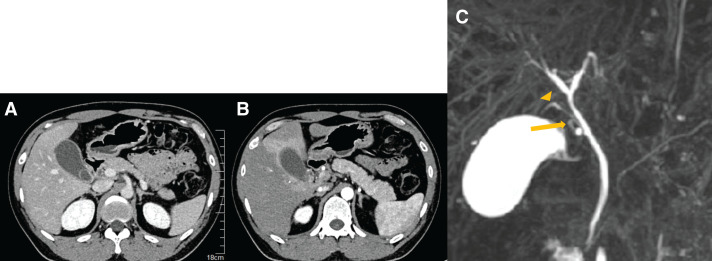
Contrast-enhanced CT scan findings at initial presentation. Contrast-enhanced abdominal CT revealed a 20-mm gallstone impacted in the gallbladder neck (**A**), with gallbladder distention and mild tension. The gallbladder wall was diffusely edematous, and early inflammatory changes were observed at the gallbladder bed (**B**). MRCP revealed that the cystic duct (arrow) drained into an AHD, specifically the bile duct from segment 6 (arrowhead) (**C**). AHD, accessory hepatic duct; MRCP, magnetic resonance cholangiopancreatography

A 35-year-old male with no significant medical history presented with a sudden onset of severe epigastric pain. He was referred to our hospital with suspected acute cholecystitis and cholelithiasis. Laboratory findings revealed a white blood cell count of 10200/μL and normal liver enzymes: aspartate aminotransferase 29 U/L, alanine aminotransferase 38 U/L, total bilirubin 1.0 mg/dL, alkaline phosphatase 107 U/L, and C-reactive protein was 0.90 mg/dL. Contrast-enhanced abdominal CT revealed a 20-mm gallstone impacted in the gallbladder neck (**Fig. 1A**), with distention, mild wall thickening, and early inflammatory changes at the gallbladder bed (**Fig. 1B**). The cystic artery originated from the right hepatic artery and passed posterior to the bile duct.

**Fig. 2:**
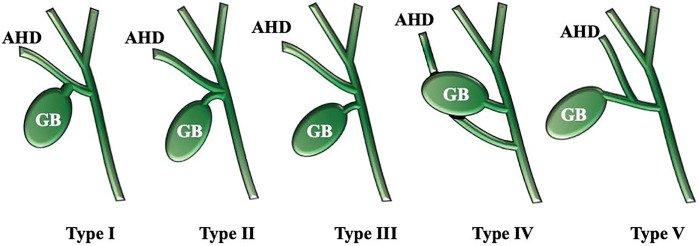
Variations of AHDs based on Hisatsugu classification. Type I: The cystic duct drains into the AHD. Type II: The cystic duct and the AHD form a common channel and drain into the common bile duct. Type III: The AHD drains into the common bile duct proximal to the cystic duct. Type IV: The AHD drains into the common bile duct distal to the cystic duct. Type V: The AHD drains into the cystic duct. AHD, accessory hepatic duct; GB, gallbladder

**Fig. 3:**
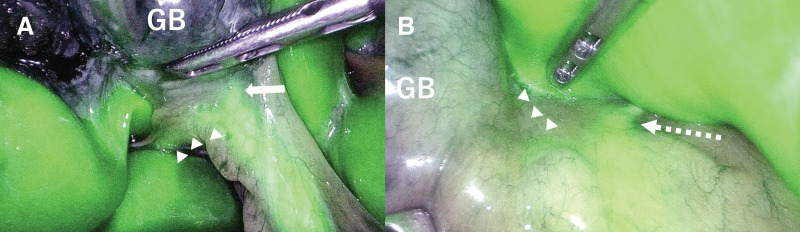
Intraoperative ICG fluorescence imaging. The B6 duct (arrowheads) was seen joining the common bile duct via the Rouviere’s sulcus, and the cystic duct (arrow) was identified as draining into B6 from a posterior approach (**A**). The course of the common hepatic duct (dashed arrow) and the B6 duct (arrowheads) was also confirmed from an anterior approach (**B**). GB, gallbladder; ICG, indocyanine green

**Fig. 4:**
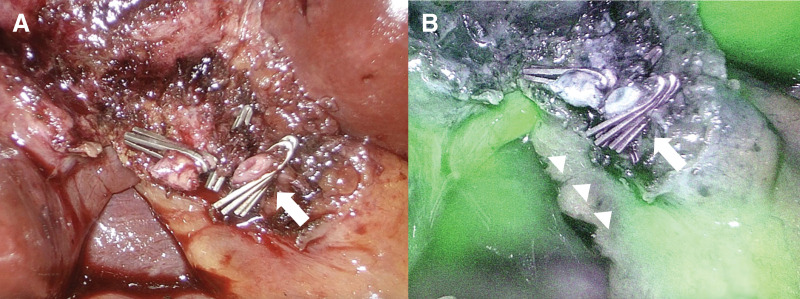
Intraoperative imaging (**A**) and ICG fluorescence imaging (**B**) after gallbladder removal. After gallbladder removal, the AHD (arrowheads), cystic duct (arrow), and common hepatic duct were confirmed again under ICG imaging. AHD, accessory hepatic duct; ICG, indocyanine green

A diagnosis of cholelithiasis with mild acute cholecystitis was made, and elective LC was planned after successful conservative management with intravenous antibiotics. Preoperative MRCP revealed the cystic duct draining into an AHD arising from segment 6 (B6), consistent with a Type I anomaly according to the Hisatsugu classification (**Figs. 1C** and **2**). The cystic duct measured approximately 15 mm from its confluence with the AHD to the gallbladder, providing a safe division length. A dose of 2.5 mg of ICG (Daiichi Sankyo, Tokyo, Japan) was injected intravenously immediately after induction of anesthesia, based on a previously reported protocol.^5)^ Fluorescence cholangiography was performed using the VISERA ELITE III system (Olympus, Hachioji, Japan), and no additional ICG injections were required during the procedure. Laparoscopy revealed a tense, distended gallbladder. Palpation confirmed a stone in the neck of the gallbladder, which was punctured, and clear bile was aspirated. The biliary anatomy was clearly visualized under ICG fluorescence imaging. The B6 duct was seen joining the common bile duct via Rouviere’s sulcus, and the cystic duct was identified as draining into B6 (**Fig. 3A**). An anterior view also confirmed this anatomy (**Fig. 3B**). Calot’s triangle was safely dissected, and the gallbladder was removed with clear visualization of the cystic duct, AHD, common bile duct, and common hepatic duct (**Fig. 4A**). Despite chronic inflammation, ICG imaging allowed safe dissection. Post-removal ICG imaging confirmed that the AHD was intact, with no injuries or strictures (**Fig. 4B**). The operative time was 3 h and 30 min, with minimal blood loss. The patient was discharged on POD 3 after an uneventful recovery.

## DISCUSSION

A right AHD, as in this case, is a rare congenital biliary anomaly, with reported incidences ranging from 0.8% to 18% in clinical studies^6–10)^ and up to 35% in autopsy series.^11)^ Several classification systems exist, including the Matsunaga classification (based on drainage level)^12)^ and the Hisatsugu classification (based on cystic duct confluence).^13)^ Hisatsugu’s Type I variant—where the cystic duct drains into the AHD—accounts for 16.8% of cases. This variant requires that the cystic duct be divided on the gallbladder side of the confluence, increasing the risk of inadvertent bile duct injury during LC.

Such anomalies significantly increase the risk of bile duct injury during LC. Severe postoperative complications can result from misidentification or inadvertent injury of an AHD.^3)^ A meta-analysis reported an 11.8-fold increase in risk in patients with biliary anomalies.^14)^ This underscores the importance of accurate preoperative imaging and intraoperative anatomical confirmation.

Safe LC requires preoperative identification of biliary anatomy, particularly in patients with suspected anatomical variants. Useful modalities for preoperative biliary evaluation include MRCP and DIC-CT. Yabe et al. reported diagnostic accuracies of 89.3% for MRCP and 100% for DIC-CT in detecting AHDs.^10)^ However, these are static assessments that lack real-time intraoperative utility. ICG fluorescence cholangiography provides dynamic, real-time visualization without bile duct cannulation or radiation exposure, making it particularly valuable in complicated or ambiguous cases.

ICG fluorescence has been widely accepted as useful for intraoperative bile duct visualization.^15–19)^ After intravenous administration, ICG is excreted into bile, allowing near-infrared imaging of the biliary tree. Previous case reports have described safe LC in patients with AHDs using ICG.^20–22)^ Tsuruda et al. identified both the AHD and cystic duct in a Type III variant using ICG.^20)^ Cases of Type I variants have also been successfully managed with this technique.^21,22)^ However, in these reports, the AHD was identified only after dissection of Calot’s triangle. In contrast, in the present case, the use of ICG fluorescence enabled clear visualization of the AHD and cystic duct even before dissection. This pre-dissection anatomical recognition may contribute to safer dissection of Calot’s triangle and help prevent bile duct injury. To the best of our knowledge, no reports exist for Types II, IV, or V. Although a few reports have described the use of fluorescence cholangiography in patients with biliary anomalies, in which the cystic and accessory ducts converge, the present case demonstrates that ICG fluorescence cholangiography allowed precise identification of the accessory duct even before Calot’s triangle dissection. This case suggests that clear visualization with ICG fluorescence imaging was likely facilitated by the mild inflammation and the relatively low amount of adipose tissue; however, ICG fluorescence cholangiography enhanced the safety of the surgical navigation in this anatomically complex setting.

While conventional intraoperative cholangiography also lowers the risk of bile duct injury,^23)^ there are associated concerns such as radiation exposure, increased operative time, and risk of injury to the bile duct during transcystic tube insertion.^22)^ These risks are particularly relevant in Types I and V anomalies, where transcystic access may inadvertently damage the AHD. Given its noninvasive nature and ability to provide real-time visualization, fluorescence cholangiography may be the more useful initial approach in cases with anatomical anomalies. ICG fluorescence imaging facilitated anatomical identification during LC in a patient with a Type I AHD. In cases where ICG fluorescence imaging does not allow clear visualization of the AHD, antegrade cholecystectomy^24)^ may serve as an alternative strategy to reduce the risk of bile duct injury. The operative time in this case was relatively long for LC. Although the intraoperative use of ICG fluorescence proceeded smoothly without technical difficulties, the presence of both cholecystitis and AHD necessitated careful dissection to avoid bile duct injury. This likely contributed to the extended operative time.

ICG fluorescence imaging also has limitations, including background fluorescence from the liver, particularly in cases with hepatic dysfunction, reduced visualization in obese or inflamed tissues, and insufficient penetration through tissues thicker than 10 mm.^25)^ In addition, impacted biliary stones can block ICG excretion and impair ductal visualization,^26)^ and although rare, allergic reactions have been reported (0.05%).^21)^ Therefore, conventional radiographic cholangiography may be more useful depending on the clinical setting.

## CONCLUSIONS

Intraoperative ICG fluorescence imaging was useful in recognizing Type I AHD and reducing the risk of bile duct injury during LC in patients with biliary anomalies. This technique offers a safe, noninvasive, real-time visualization method that enhances surgical navigation in anatomically complex settings.

## DECLARATIONS

### Availability of data and materials

The authors declare that all data within this article are available upon reasonable request. The data supporting the findings of this study are available from the corresponding author upon reasonable request.

## References

[1] Warchałowski Ł, Łuszczki E, Bartosiewicz A, et al. The analysis of risk factors in the conversion from laparoscopic to open cholecystectomy. Int J Environ Res Public Health 2020; 17: 7571. 33080991 10.3390/ijerph17207571 PMC7588875

[2] Zidan MHE, Seif-Eldeen M, Ghazal AA, et al. Post-cholecystectomy bile duct injuries: a retrospective cohort study. BMC Surg 2024; 24: 8. 38172774 10.1186/s12893-023-02301-2 PMC10765830

[3] Vasiliadis K, Moschou E, Papaioannou S, et al. Isolated aberrant right cysticohepatic duct injury during laparoscopic cholecystectomy: evaluation and treatment challenges of a severe postoperative complication associated with an extremely rare anatomical variant. Ann Hepatobiliary Pancreat Surg 2020; 24: 221–7. 32457271 10.14701/ahbps.2020.24.2.221 PMC7271109

[4] Cassinotti E, Al-Taher M, Antoniou SA, et al. European Association for Endoscopic Surgery (EAES) consensus on indocyanine green (ICG) fluorescence-guided surgery. Surg Endosc 2023; 37: 1629–48. 36781468 10.1007/s00464-023-09928-5 PMC10017637

[5] Pardo Aranda F, Gené Škrabec C, López-Sánchez J, et al. Indocyanine green (ICG) fluorescent cholangiography in laparoscopic cholecystectomy: simplifying time and dose. Dig Liver Dis 2023; 55: 249–53. 36404235 10.1016/j.dld.2022.10.023

[6] Okada K, Tamio T, Sakuramoto K, et al. Studies with ERCP in cases with anomalies of biliary tract, with special references of variation of cystic duct (in Japanese). Jpn J Gastroenterol Surg 1981; 14: 1197–203.

[7] Hayes MA, Goldenberg IS, Bishop CC. The developmental basis for bile duct anomalies. Surg Gynecol Obstet 1958; 107: 447–56. 13580794

[8] Michels NA. Newer anatomy of the liver and its variant blood supply and collateral circulation. Am J Surg 1966; 112: 337–47. 5917302 10.1016/0002-9610(66)90201-7

[9] Sureka B, Bansal K, Patidar Y, et al. Magnetic resonance cholangiographic evaluation of intrahepatic and extrahepatic bile duct variations. Indian J Radiol Imaging 2016; 26: 22–32. 27081220 10.4103/0971-3026.178283 PMC4813070

[10] Yabe S, Nakagawa T, Okumura K, et al. Preoperative evaluation and management of accessory hepatic ducts for cholecystectomy (in Japanese with English abstract). Jpn J Gastroenterol Surg 2020; 53: 399–408.

[11] Healey JE Jr., Schroy PC. Anatomy of the biliary ducts within the human liver: analysis of the prevailing pattern of branchings and the major variations of the biliary ducts. AMA Arch Surg 1953; 66: 599–616. 13039731 10.1001/archsurg.1953.01260030616008

[12] Matsunaga A, Tokunaga S, Takeda M, et al. A clinical study on the accessary hepatic duct (in Japanese). J Jpn Surg Soc 1989; 22: 65–71.

[13] Hisatsugu T, Yamamoto H, Igimi H, et al. Anomaly of bile duct including aberrant right hepatic duct with cholecystolithiasis (in Japanese). J Adult Dis 1974; 4: 581–6.

[14] Yang S, Hu S, Gu X, et al. Analysis of risk factors for bile duct injury in laparoscopic cholecystectomy in China: a systematic review and meta-analysis. Medicine (Baltimore) 2022; 101: e30365. 36123939 10.1097/MD.0000000000030365 PMC9478294

[15] Ishizawa T, Tamura S, Masuda K, et al. Intraoperative fluorescent cholangiography using indocyanine green: a biliary road map for safe surgery. J Am Coll Surg 2009; 208: e1–4. 10.1016/j.jamcollsurg.2008.09.024 19228492

[16] Ishizawa T, Bandai Y, Kokudo N. Fluorescent cholangiography using indocyanine green for laparoscopic cholecystectomy: an initial experience. Arch Surg 2009; 144: 381–2. 19380655 10.1001/archsurg.2009.9

[17] Ishizawa T, Bandai Y, Ijichi M, et al. Fluorescent cholangiography illuminating the biliary tree during laparoscopic cholecystectomy. Br J Surg 2010; 97: 1369–77. 20623766 10.1002/bjs.7125

[18] Kono Y, Ishizawa T, Tani K, et al. Techniques of fluorescence cholangiography during laparoscopic cholecystectomy for better delineation of the bile duct anatomy. Medicine (Baltimore) 2015; 94: e1005. 26107666 10.1097/MD.0000000000001005 PMC4504575

[19] Sheffield KM, Han Y, Kuo YF, et al. Variation in the use of intraoperative cholangiography during cholecystectomy. J Am Coll Surg 2012; 214: 668–79; discussion 679-81. 22366491 10.1016/j.jamcollsurg.2011.12.033 PMC3319194

[20] Tsuruda Y, Okumura H, Setoyama T, et al. Laparoscopic cholecystectomy with aberrant bile duct detected by intraoperative fluorescent cholangiography concomitant with angiography: a case report. Int J Surg Case Rep 2018; 51: 14–6. 30130667 10.1016/j.ijscr.2018.08.009 PMC6104581

[21] Yoo D. Laparoscopic cholecystectomy for a gallbladder with a short cystic duct draining to the accessory right anterior hepatic duct using indocyanine green fluorescence imaging: a case report. Int J Surg Case Rep 2024; 121: 110014. 38981297 10.1016/j.ijscr.2024.110014 PMC11294690

[22] Kinoshita M, Watanabe S, Mizojiri G, et al. Fluorescence cholangiography for detection of a cystic duct drained into an accessory hepatic duct: a case report. Int J Surg Case Rep 2023; 102: 107808. 36495753 10.1016/j.ijscr.2022.107808 PMC9730159

[23] Flum DR, Dellinger EP, Cheadle A, et al. Intraoperative cholangiography and risk of common bile duct injury during cholecystectomy. JAMA 2003; 289: 1639–44. 12672731 10.1001/jama.289.13.1639

[24] Tartaglia N, Cianci P, Di Lascia A, et al. Laparoscopic antegrade cholecystectomy: a standard procedure? Open Med (Wars) 2016; 11: 429–32. 28352832 10.1515/med-2016-0078 PMC5329865

[25] Symeonidis S, Mantzoros I, Anestiadou E, et al. Biliary anatomy visualization and surgeon satisfaction using standard cholangiography versus indocyanine green fluorescent cholangiography during elective laparoscopic cholecystectomy: a randomized controlled trial. J Clin Med 2024; 13: 864. 38337557 10.3390/jcm13030864 PMC10856121

[26] Wang ZH, Yan S, Wang R, et al. Clinical application of indocyanine green fluorescence imaging in laparoscopic cholecystectomy with common bile duct exploration and J-Tube drainage. World J Gastrointest Surg 2025; 17: 99495. 39872786 10.4240/wjgs.v17.i1.99495 PMC11757186

